# Reconfigurable Local Photoluminescence of Atomically-Thin Semiconductors via Ferroelectric-Assisted Effects

**DOI:** 10.3390/nano9111620

**Published:** 2019-11-15

**Authors:** Changhyun Ko

**Affiliations:** 1 Department of Applied Physics, College of Engineering, Sookmyung Women’s University, Seoul 04310, Korea; cko@sookmyung.ac.kr; Tel.: +82-2-6325-3184; 2 Institute of Advanced Materials and Systems, Sookmyung Women’s University, Seoul 04310, Korea

**Keywords:** transition metal dichalcogenides, molybdenum disulfide, two-dimensional materials, ferroelectrics, photoluminescence

## Abstract

Combining a pair of materials of different structural dimensions and functional properties into a hybrid material system may realize unprecedented multi-functional device applications. Especially, two-dimensional (2D) materials are suitable for being incorporated into the heterostructures due to their colossal area-to-volume ratio, excellent flexibility, and high sensitivity to interfacial and surface interactions. Semiconducting molybdenum disulfide (MoS_2_), one of the well-studied layered materials, has a direct band gap as one molecular layer and hence, is expected to be one of the promising key materials for next-generation optoelectronics. Here, using lateral 2D/3D heterostructures composed of MoS_2_ monolayers and nanoscale inorganic ferroelectric thin films, reversibly tunable photoluminescence has been demonstrated at the microscale to be over 200% upon ferroelectric polarization reversal by using nanoscale conductive atomic force microscopy tips. Also, significant ferroelectric-assisted modulation in electrical properties has been achieved from field-effect transistor devices based on the 2D/3D heterostructrues. Moreover, it was also shown that the MoS_2_ monolayer can be an effective electric field barrier in spite of its sub-nanometer thickness. These results would be of close relevance to exploring novel applications in the fields of optoelectronics and sensor technology.

## 1. Introduction

Semiconducting layered transition metal dichalcogenides (TMDs) have been studied widely due to their strikingly interesting electronic and optoelectronic aspects unveiled in the two-dimensional (2D) limit including thickness-dependent bandgap, indirect-to-direct transitions of band structures, deeply-bound excitonic states, environment-sensitive characteristics, and so on [1,2,3,4,5]. Beyond fundamental interests, diverse device applications have also been realized from elementary field-effect transistors (FETs) and light-emitting diodes to complicated microprocessor structures as well as functional device components which enable memory, sensing, and resistive switching effects [6,7,8,9,10]. This ever-rising eagerness for the 2D TMD-based applications stems from a strong demand to replace Si-based components exclusively employed in the modern nano-electronics with the class of 2D semiconductors to overcome the fundamental scaling limitations, boost up integration density, and more innovatively, invent novel device platforms such as ultrathin flexible electronics [11].

However, despite the continual progress in this field, it is still challenging to incorporate 2D semiconductors into commercial products since material synthesis and device fabrication processes with 2D materials are currently not very cost-efficient and limited in scalability. Also, their operational device parameters such as carrier mobility and power consumption are expected to be inferior, out of laboratory, to those of contemporary devices which have been optimized for several decades [12]. Therefore, as promising alternative routes, diverse approaches have been made to assemble 2D semiconductors and a variety of 3D thin films structures which are very suitable for contemporary device fabrication infrastructure including classical dielectrics, semiconducting layered crystals, strongly correlated oxides, ferromagnetic materials, and ferroelectric (FE) thin epitaxies [8,12,13,14,15,16,17,18]. In these 2D/3D assemblies, in addition to size scaling benefits naturally given by introducing 2D materials, interfacial interference effects or thin films’ functional attributes or both can be instilled into the 2D counterpart leading to achieving unique synergetic functionalities [12].

FE material systems where the electric polarization is built up spontaneously and can be flipped to the opposite direction abruptly by electrical stimulus have been considered intensively for various applications such as nonvolatile memory devices, photodetectors, water splitting, and photocatalysts [19,20,21,22,23]. Most of all, the ferroelectric FET (FeFET) where a dielectric layer in the conventional FET is replaced by a FE thin film has been considered as a promising candidate of next-generation memory with an advantage of nondestructive readout operation [19]. In the FeFET, the channel current can be modulated by ferroelectric gating. Due to the spontaneous polarization created in the FE thin film, the current can be maintained even after the removal of the gate voltage. More importantly, the FeFET structure can be realized in 2D/3D heterostructures simply by positioning 2D semiconductors as current channels on FE thin films [8,19]. Previously, the author of this paper and colleagues fabricated high-performance 2D/3D FeFET memory devices based on 2D MoS_2_ and WSe_2_ layers prepared by mechanical exfoliation from corresponding single crystals and lead zirconate titanate (PZT) epitaxial FE thin films [8]. More interestingly, the reversible nonvolatile photoluminescence (PL) modulation was also observed from the monolayer MoS_2_ (ML-MoS_2_) on the PZT thin film [8]. More recently, the ferroelectric control of PL was also demonstrated on other types of 2D TMDs, mechanically-exfoliated ML-MoSe_2_ and ML-WSe_2_ on the domain-engineered lithium niobate surface by B. Wen. et al. [24]. Also, M. Si. et al. reported fully-layered FeFET structures where 2D MoS_2_ layers are interfaced with ferrielectric CuInP_2_S_6_ layered crystals [25].

As another type of FE-based device, the ferroelectric tunnel junction (FTJ) where a FE thin film is typically sandwiched by two metallic electrodes shows out-of-plane current ON/OFF switching via the change in band structure upon polarization reversal [26,27]. T. Li et al. demonstrated FTJs in the use of conductive atomic force microscopy (CAFM) technique on the 2D MoS_2_/thin film BaTiO_3_ heterostructures in which the top electrodes are few-layer MoS_2_ layers grown by chemical vapor deposition (CVD) [26]. More recently, A. Lipatov et al., by local access to nanoscale FE domains via CAFM, realized programmable 1D current paths on CVD-grown ML-MoS_2_ flakes using ferroelectric effects [27]. However, in this case, the in-plane current modulation shows the opposite trend to that observed in the typical FeFET with respect to the polarization direction. Although the authors argue that the in-plane current and out-of-plane current measured in the FTJ geometry on the identical devices should be correlated somehow, to elucidate mechanisms clearly, it would be necessary to investigate the FE effects into both device characteristics and optical properties [2,8,24,26,27]. Further, in the case of CVD-grown TMD MLs, inherent defective structures usually exist and hence, in comparison to the mechanically exfoliated MLs, the defect-sensitive characteristics should be carefully considered to fully understand the modulation of electrical and optical properties of TMD MLs driven by ferroelectric effects [28,29,30,31].

In this work, exploiting heterostructures of ML-MoS_2_/FE thin epitaxy, the reversible modulation of nonvolatile PL, has been demonstrated at the microscale via ferroelectric-assisted effects. The electric field was applied directly through the heterostructures with both atomic formic microscopy (AFM) probe tips and electrical back-gated devices. Moreover, the polarization-induced manipulation in electrical characteristics was also realized on the FET devices based on the heterostructures along with simultaneous control of optical properties of the ML-MoS_2_ channels. Lastly, in the same ML-MoS_2_/FE geometry, I have also found that ML-MoS_2_ sheets, of only sub-nanometer thickness, may play a role of an electric field barrier properly. The ML-MoS_2_ layers were grown by the CVD method. Considering the recent progress in the CVD growth of 2D materials, this work would be relevant to the scalable and controllable fabrication of novel multi-dimensional devices [32]. As the FE components, two different inorganic FE materials were employed: PZT and BiFeO_3_ (BFO) epitaxial thin films. The former has been studied widely for device applications due to their ultrafast dipole dynamics, good thermal and mechanical stability, and high dielectric breakdown limit [8,19,33]. Moreover, the latter produces the stronger polarization field which may lead to better device functionality [34,35].

## 2. Materials and Methods

### 2.1. MoS_2_ Monolayer Growth

Triangular-shaped ML-MoS_2_ flakes were grown on 100-nm-thick SiO_2_/Si substrates via CVD after cleansing the substrates with Piranha solution and deionized (DI) water in a series. The ML-MoS_2_ growth was carried out with the substrates face-down on an alumina crucible containing 3 mg of MoO_3_ powder while the other crucible with S powder was located closer to the gas source than that with MoO_3_ source. Initially, the furnace tube was purged by flowing ultrahigh purity N_2_ gas at a flow rate of 500 SCCM (standard cubic centimeters per minute) for 10 min. Then, the heating process was performed in two steps: (1) The system was heated up to 300°C for ~10 min flowing N_2_ at 100 SCCM; and (2) the temperature was increased up to 700 °C above the boiling temperature of S (~450 °C) within 15 min with N_2_ gas flow of 5 SCCM and these conditions were sustained for 3 min. The furnace power was then shut down. Once the temperature reached 680 °C, the furnace was slightly opened and at 550 °C, the growth tube was detached entirely from the furnace to quench the samples. S vapor was supplied to the samples continuously even during the cooling step at a flow rate in the range of 2 to 5 SCCM to preserve the sample quality.

### 2.2. Ferroelectric Thin Film Synthesis and Heterostructure Fabrication

In this study, PZT (Pb[Zr_0.2_Ti_0.8_]O_3_) and BFO (BiFeO_3_) thin films were employed as the FE components of the ML-MoS_2_/FE heterostructures. PZT (or BFO) thin films were grown epitaxially with a thickness of ~500 nm (or ~100 nm) on (001) STO (SrTiO_3_) single-crystal substrates coated with SRO (SrRuO_3_) layers by pulsed-laser deposition (PLD) utilizing a KrF laser (wavelength: ~248 nm) under a substrate temperature of 630 °C (or 690 °C), respectively. The buffer layer SRO was deposited at 740 °C also by PLD. During the deposition, a small amount of O_2_ gas was supplied into the chamber maintaining the total pressure at ~100 mTorr. Subsequent to the deposition processes, the films were cooled down to room temperature at an O_2_ pressure of 500 Torr with a cooling rate of 5 °C/min. The various thin film evaluations showed that the FE films used in this work have high quality elsewhere [8,29]. Then, the ML-MoS_2_/FE heterostructures were constructed by transferring the as-grown MoS_2_ flakes from SiO_2_/Si substrates onto the FE thin films surface using polydimethylsiloxane (PDMS) films. The ML-MoS_2_ flakes were detached from the SiO_2_/Si substrates by wet etching with KOH solution. Also, before the transfer process, the surfaces of FE thin films were cleaned properly by gentle oxygen plasma etching.

### 2.3. Characterization of MoS_2_ Monolayers and Heterostructures

Micro-PL and Raman experiments were conducted using objective lenses on a Renishaw micro-Raman/PL system (Gloucestershire, UK) operated with an excitation laser of ~488 nm wavelength in ambient conditions. The laser power was set properly in the range from 0.1 to 1 μW depending on the laser scan time to avoid any damage on the ML-MoS_2_ sheets as well as FE thin films. The focused laser beam covers an area of ~6 μm^2^. Out-of-plane piezoresponse force microscopy (PFM) was carried out on Veeco Multiprobe system (Plainview, NY, USA) to visualize the domain structure which is controllable by an electric field at the sub-microscale in air. FE domains were manipulated by applying a voltage in the range of −12 V to +12 V through conventional conductive AFM tips. Contact mode AFM was also performed simultaneously to obtain surface topography images of ML-MoS_2_ flakes on FE thin films.

### 2.4. Device Fabrication and Electrical Characterization

Conventional electron beam lithography was processed to lay out the electrode patterns. Ti/Au metal contacts with 10 nm/70 nm thicknesses were fabricated by e-beam evaporation and subsequent lift-off process. Back-gated FET measurements were conducted on a standard probe station equipped with two Keithley 617 programmable electrometers (Cleveland, OH, USA) which were designed to supply voltage to devices and to measure the source-drain and leakage currents separately in the ambient conditions.

## 3. Results and Discussion

### 3.1. 2D MoS_2_/FE Heterostructure Preparation

**Figure 1:**
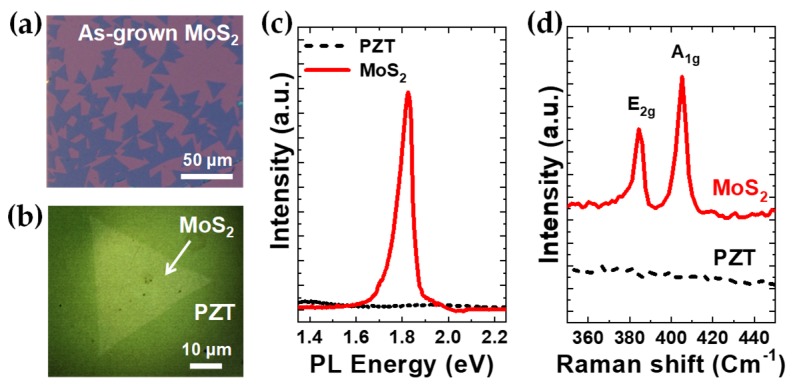
ML-MoS_2_/PZT heterostructure preparation and optical characterization. Optical microscopy images of (**a**) as-grown ML-MoS_2_ flakes on a SiO_2_/Si substrate and (**b**) a representative ML-MoS_2_ flake wet-transferred on a PZT thin film surface. (**c**) PL and (**d**) Raman spectra measured from the ML-MoS_2_ flake displayed in (**a**) and the bare PZT surface as a reference.

Figure 1a shows a group of MoS_2_ flakes grown on a SiO_2_/Si substrate with a triangular shape, as typically observed on the family of CVD-grown TMD MLs. By the wet-transfer method, the MoS_2_ sheets were micro-positioned on the clean surface area of ~500-nm-thick PZT thin films solidly, as shown in the optical microscopy image of Figure 1b. As summarized in Figure 1c,d, the optical characterization was conducted to determine the layer number of the CVD-grown MoS_2_ flakes as well as to evaluate their quality. In Figure 1c, the PL spectrum of the MoS_2_ flake clearly indicates the direct band-gap nature with the strong PL peak at ~1.84 eV verifying that the MoS_2_ flakes used in this work are MLs [1,2,5,8]. Based on the Raman spectrum in Figure 1d, ML-MoS_2_ sheets on PZT surface are not strained or damaged significantly through the wet-transfer process. Optical characterization was also performed on the ML-MoS_2_/BFO heterostructures (see Supplementary Figure S1).

### 3.2. Reversible Ferroelectric Modulation of Local Photoluminescence

Many semiconducting members of the TMD group, such as MoS_2_, WS_2_, WSe_2_, MoSe_2_, etc., show direct band gaps with a single molecular layer while indirect band gap structures are observed in their bulk counterparts [1,5,8,24]. Therefore, through the radiative exciton recombination, strong PL emission is observed in the direct-gap MLs. Further, the optoelectronic characteristics are strongly affected by the existence of quasiparticles including neutral excitons (X) of e-h pairs and charged excitons, also called trions, (X^−^ or X^+^) of e-e-h or e-h-h complexes, respectively, even at room temperature in contrast with conventional semiconductors. Therefore, the PL emissions of ML-MoS_2_ can be modulated in terms of peak intensity and position, controlling the concentrations and types of the species. Up to now, numerous approaches have been demonstrated for altering the PL emissions: electrostatic charging, chemical treatment, physisorption, point defect formation, substitutional doping, and so on [2,5,24,31,36,37]. In this work, using spontaneous polarization of FE materials, the densities of excitons and trions in ML-MoS_2_ were controlled in a nonvolatile way and eventually, optoelectronic memory effects were realized in two-different ways: (1) local gating through conductive AFM tips, called the poling process, and (2) electrical back-gating with patterned metal electrodes.

**Figure 2:**
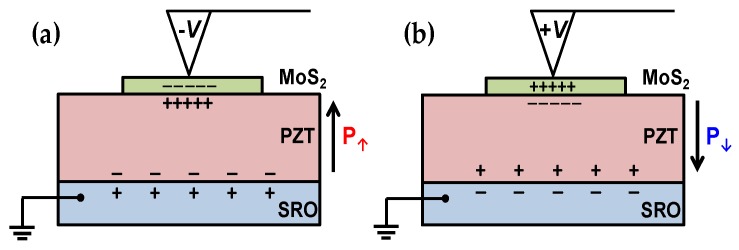
Schematic of the poling process on AFM. (**a**) Up-polarized (P_↑_) and (**b**) down-polarized (P_↓_) states are achieved when a PZT thin films are applied by negative and positive *V_P_* above the threshold voltage for polarization reversal using a conductive AFM tip, respectively. During the poling process, a metallic SRO layer is grounded electrically.

Figure 2a,b shows schematically how the poling process is performed by applying poling voltage (*V_P_*) using an AFM tip across a PZT thin film sandwiched by a ML-MoS_2_ sheet and a metallic SRO layer which play roles as electrical pads on each side. When the *V_P_* is large enough to induce polarization reversal above the coercive field of the PZT thin film, the up-polarized (P_↑_) and down-polarized (P_↓_) states are built up with the negative and positive *V_P_*, respectively, and the polarization states are maintained even after the detachment of the AFM tip due to spontaneous polarization. Based on the schematics, it is expected that in the n-type semiconductor ML-MoS_2_ flakes, the concentration of the majority carrier, electron, would be enhanced in the P_↑_ state locally around the contact area of the AFM tip, while in the P_↓_ state, electrons would be depleted.

**Figure 3:**
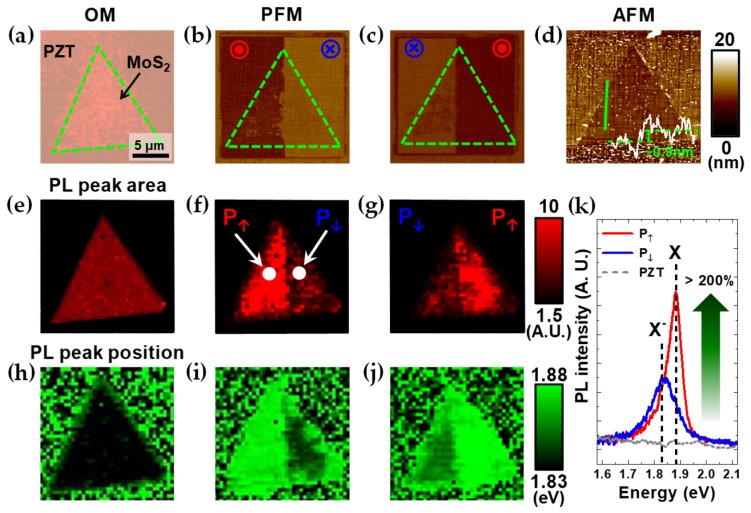
Poling effects on the ML-MoS_2_/PZT heterostructure. (**a**) Optical microscopy (OM) image of a ML-MoS_2_ flake with a scale bar which works for all the other images. PFM images of (**b**) the ML-MoS_2_ flake on the PZT thin film whose left and right half areas are polarized in the P_↑_ and P_↓_ states by the poling process with the *V_P_* of −12 V and +12 V, respectively, and (**c**) vice versa. (**d**) Topography AFM image of the ML-MoS_2_ flake simultaneously obtained with the PFM image including the representative height profile for the corresponding green line, verifying the thickness of ML-MoS_2_ flake. PL peak area maps of the identical ML-MoS_2_ flake in (**e**–**g**) were scanned before poling and after the poling processes of (**b**,**c**), in order. (**h**–**j**) PL peak position maps displayed in the same sequence as in (**e**–**g**). (**k**) PL spectra measured from the spots of P_↑_ and P_↓_ regions marked in (**f**) along with that of the bare PZT as a reference. X and X^−^ denote the emissions of neutral and negatively-charged excitons, respectively.

Figure 3 summarizes how the PL emission of a ML-MoS_2_ flake on a PZT thin film can be controlled ferroelectrically by the poling process. The non-volatile PL feature given by the poling process was observed to not be degraded that much up to several days in the air. Figure 3a,d are optical microscopy and AFM topography images taken from the ML-MoS_2_ flake transferred on the PZT surface. The representative height profile embedded in Figure 3d verifies the thickness of ML-MoS_2_ while the surface roughness reflects the inherent domain structure of the PZT thin film [8]. Figure 3b,c include the PFM images of the ML-MoS_2_ flake on the PZT film, in which half of its area is in the P_↑_ state and the rest is in the opposite P_↓_ state. From the PL peak area and position maps in Figure 3e–j, the PL intensity of ML-MoS_2_ is stronger by more than 200% and the PL peak position is more blue-shifted in the P_↑_ state than in the P_↓_. The difference in the PL characteristics can be more clearly observed from the PL spectra measured from the spots of the ML-MoS_2_ flake in the P_↑_ and P_↓_ regimes, selectively as shown in Figure 3k. By deconvoluting the PL peaks into two Lorentzian peaks located at ~1.88 eV and ~1.84 eV corresponding to the transitions of X and X^−^, respectively, the relative contributions of the two different species can be analyzed for each case. While the PL peak is contributed to from both emissions in the P_↑_ state, the emission of X is suppressed almost completely in the P_↓_ state. These results show that the control of carrier concentration via FE gating allows PL emissions to be modulated reversibly via exciton-trion transition [8,24]. The same trend was also observed in the ML-MoS_2_/BFO heterostructures (see Supplementary Figure S2). Further, the microscale PL modulation has been demonstrated more clearly with a stripe poling pattern of a domain-engineered BFO surface (see Supplementary Figure S3). It is worthy to note that, considering the nanoscale size of the AFM tip, the PL modulation can be confined even down to the nanoscale by this approach, as the electrical properties were tuned on ML-MoS_2_/PZT at the nanoscale by A. Lipatov and his colleagues [27]. However, the trend of PL modulation observed here is opposite to that predicted from the schematics of the poling process in Figure 2 and also that of the previously reported ML-TMD/FE heterostructures whose ML-TMD parts were prepared by mechanical exfoliation from single crystals [8,24]. This inconsistency will be discussed in detail later.

**Figure 4:**
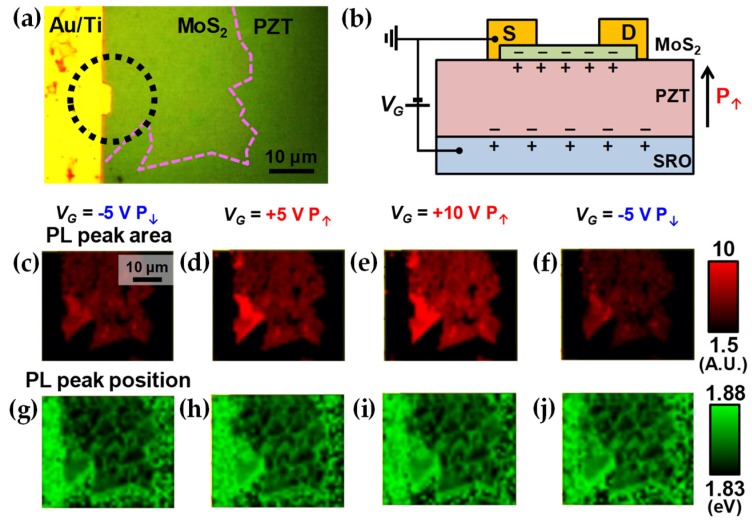
Electrical back-gated experiments on the ML-MoS_2_/PZT heterostructures. (**a**) Optical microscopy image of a ML-MoS_2_ sheet with a metal electrode. (**b**) Schematics of back-gating experiment. In this experiment, no voltage is applied between the source and drain. (**c**–**f**) Set of images of the PL peak area measured after back gating with *V_G_* of −5 V, +5 V, +10 V, and again −5 V in order. (**g**–**j**) The set of images of PL peak position measured in a series in the same order of (**c**–**f**).

The same trend of polarization-dependent PL has also been observed by electrostatic gating by which the control of polarization of PZT thin films can be performed more efficiently and also more safely than the poling process with the direct contact of AFM tips. As shown in Figure 4a, the metal electrode was fabricated on a ML-MoS_2_ flake. Figure 4b shows schematically how a PZT thin film can be up-polarized by back-gating with a positive gate voltage (*V_G_*) from a bottom electrode of a SRO thin layer. However, the ML-MoS_2_ flake seems to be damaged partly through the fabrication process; from the PL peak area maps in Figure 4c–f scanned after the *V_G_* is removed, it can be seen clearly that the PL intensity can be modulated reversibly in a nonvolatile way between the P_↑_ and P_↓_ states. The PL modulation is observed to be intensified particularly near the bump inside the dashed circle in Figure 4a where the electric field is focused strongly. Consistently with the results of the first AFM experiment, the PL intensity was observed to be higher in the P_↑_ state than in the opposite P_↓_ state. Also, as can be checked in Figure 4g–j, as the PL intensity increases with strengthening up-polarization, the PL peak is blue-shifted slightly, which is probably due to the enhancement of emissions by X.

### 3.3. Electrical Transport Characterization

**Figure 5:**
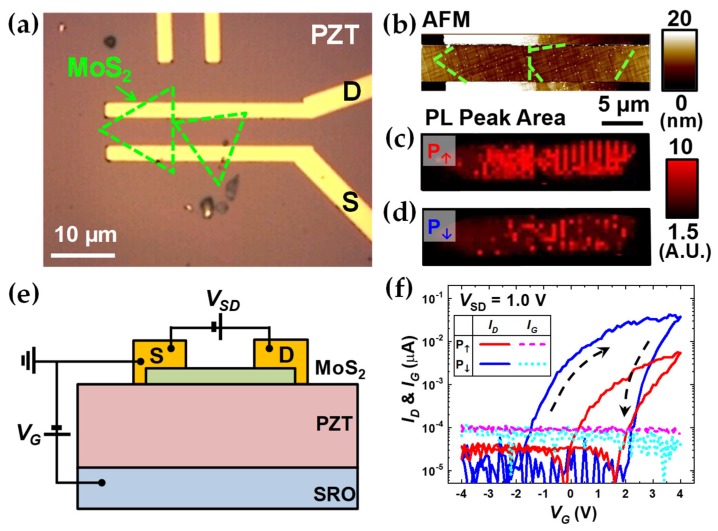
Electrical transport characterization of the ML-MoS_2_/PZT heterostructures. (**a**) Optical microscopy image and (**b**) topography AFM image of a device with a channel based on two ML-MoS_2_ sheets and metal electrodes on a PZT thin film. PL peak area maps of (**c**,**d**) were scanned after the poling process with *V_P_* of −12 V and +12 V, respectively. (**e**) Device schematics for FET measurements with the circuit. (**f**) FET characteristic curves of drain current (*I_D_*) vs. gate voltage (*V_G_*) measured at the source-drain voltage (*V_SD_*) of 1.0 V in the P_↑_ and P_↓_ states, respectively. The leakage current (*I_G_*) vs. *V_G_* are also plotted as dashed lines for both states. Note that the absolute values were taken for *I_D_* and *I_G_*.

To understand the underlying mechanisms of the PL modulation induced by FE polarization deeply, the combined experiments of optical and electrical measurements are also carried out on a FET device based on two ML-MoS_2_ flakes. The optical microscopy and AFM topography images of the device are shown in Figure 5a,b, respectively. After each poling process, both PL maps and FET characteristic curves were acquired. Figure 5c,d shows that the PL of the ML-MoS_2_ flakes embedded in the device can also be modulated by the poling process properly. To achieve the P_↑_ and P_↓_ states, the area including the ML-MoS_2_ region was scanned, applying *V_P_* of −12 V and +12 V with a AFM tip, respectively. Figure 5e shows schematics of the device with the circuit for the FET measurements. As shown in the drain current (*I_D_*) vs. *V_G_* plots in Figure 5f, in the both the P_↑_ and P_↓_ states, the typical n-type FET characteristics are observed with a large hysteresis probably due to the environmental effects [38]. Also, it can be seen that the very high dielectric constant of the PZT enables low-power FET operation with very small *V_G_* [8,39]. The conduction level is higher in the P_↓_ state than the P_↑_ state by almost an order of magnitude implying that the electron concentration is also higher in the P_↓_ state, which is consistent with the analysis with the PL spectra. In addition, the variation of leakage current (*I_G_*) with the polarization direction is likely to be driven by the polarization-induced band structure change across the ML-MoS_2_/PZT junction [26].

Now, based on all these results, it will be discussed why the trends of FE effects observed in this work are contrast to those of conventional FeFETs [8,19,27]. As in the first scenario, positively-charged exciton X^+^, which is expected to have similar PL characteristics to those of negatively-charged exciton X^−^, may be excited dominantly in the P_↓_ state as inversion occurs in the ML-MoS_2_ flake [5]. While A. Lipatov et al. argued that the majority carriers of ML-MoS_2_ on PZT or BTO would be holes, in this case, electrons should be majority carriers from the n-type conduction of the FET devices as shown in Figure 5f [27]. The n-type conduction is even stronger in the P_↓_ state than that in the P_↑_ state, ruling out the possibility of the dominance of X^+^. Moreover, from the FET measurements, only clockwise FET hysteresis loops were observed. Typical counterclockwise operation for FeFETs caused by spontaneous polarization and abrupt charging/discharging upon polarization reversal were not observed even from the FET characterization in the wider *V_G_* range well above the threshold voltage of PZT thin films of ~3–4 V (see Supplementary Figure S4) [8,19].

Along the van der Waals interfaces between the CVD-grown ML-MoS_2_ layers and the FE surface, the interfacial traps and contaminants can be formed during the wet-transfer process for fabricating heterostructures and may mitigate the interaction across the 2D/FE interface [14]. The significant hysteresis of FET characteristic loops indicates a strong influence of environmental factors such as molecular adsorption, humidity, interfacial traps, and so on [14,38,40]. Further, maybe due to the complicated interplay among inherent defects of the CVD-grown ML-MoS_2_ and the interfacial traps and contaminants, abnormal defect-related interactions coupled with FE polarization may be activated. For example, the ionic species of the interfacial trapped water layer are likely to be injected into or removed from the ML-MoS_2_ surface through the reversible electrochemical process induced by FE polarization [21,41]. It is known that O_2_ or water molecules take away electrons from ML-MoS_2_ more actively around point defects such as S vacancies [5,29,31]. Accordingly, the ionic species seem to charge or discharge the ML-MoS_2_ interacting with the point defects differently depending on the polarization state. Moreover, the ambient molecules such as O_2_ and water vapor may be involved in the modulation of carrier concentrations in ML-MoS_2_. From the results of optical and electrical characterization, the charge transfer from ML-MoS_2_ to trapped ionic species is likely to be more active in the P_↑_ state leading to a decrease in the carrier concentration and eventually, the enhancement of emissions of X. To verify the trap effects as well as facilitate the FE effects fully, trap-free van der Waals interfaces should be achieved possibly by assembling the heterostructures using the dry-transfer method in the inert gas environment [14]. Therefore, the ML MoS_2_/FE heterostructures may also have potential to be applicable to ionic sensors besides optoelectronic devices.

### 3.4. Atomically-Thin Electric Field Barrier

**Figure 6:**
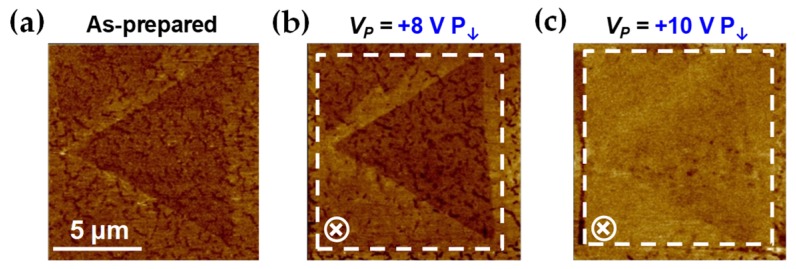
The ultrathin electric field shield. PFM images of a ML-MoS_2_ flake on a BFO thin film scanned (**a**) as-prepared before poling and after poling with the *V_P_* of (**b**) +8 V and (**c**) +10 V, respectively. Up to *V_P_* of +8 V, the area of the BFO thin film beneath the ML-MoS_2_ flake was not poled well, indicating that a ML-MoS_2_ flake prevents field penetration into the BFO thin film.

Lastly, ML-MoS_2_ flakes have been evaluated for whether they work well as electric field barriers using 2D/FE hybrid platforms under the poling process in air or not. Figure 6 shows that ML-MoS_2_ prevents electric field penetration into a BFO thin film quite well considering its sub-nanometer thickness. While ML-MoS_2_ is quite conductive with the Fermi energy level located very closely to the conduction band edge in a vacuum, it becomes less conductive in air along with the shift of the Fermi energy toward the midgap energy level via interacting with ambient gas such as O_2_ and water vapor [40]. Figure 6a shows that the PFM image scanned from just after the ML-MoS_2_ flake was transferred on the unpoled region of the BFO thin film which shows the irregular polarization pattern of the pristine BFO thin film. Since the ML-MoS_2_ sheet is ultrathin and almost transparent to the piezoelectric response, the pattern can be seen through the ML-MoS_2_ flake clearly. Up to *V_P_* of +8 V, the region of the BFO thin film underneath the ML-MoS_2_ flake was not poled well, indicating that the ML-MoS_2_ can block the electric field significantly. The ML-MoS_2_ regime was down-polarized finally after poling with *V_P_* of +10 V. The electric field was observed to be shielded during the poling process to achieve the opposite up-polarized states on the same heterostructure. Moreover, also in the case of the ML-MoS_2_/PZT system, similar electric field screening effects were observed (see Supplementary Figure S5). Now, it is clearly understood why PL modulation can be achieved upon polarization reversal only when the application of *V_P_* (or *V_G_*) is well above the threshold voltage of ~3–4 V, which is determined by the coercive field of the FE films.

## 4. Conclusions

In this research, the alteration of nonvolatile PL over 200% has been achieved upon polarization reversal along with large modulation in electrical properties in the use of lateral ML-MoS_2_/FE heterostructures. Based on comprehensive experiments and analyses, the spontaneous polarization of the FE thin films seems to affect the optoelectronic behaviors of ML-MoS_2_ indirectly via reversible electrochemical processes among interfacial traps, air molecules, and structural imperfections. In addition, in the same geometry, it was also shown that MoS_2_ can shield the electric field effectively even with sub-nanometer thickness in ambient conditions.

## Supplementary Materials

The following are available online at https://www.mdpi.com/2079-4991/9/11/1620/s1, Figure S1: Optical characterization of the ML-MoS_2_/BFO heterostructure. Figure S2: Poling effects on the ML-MoS_2_/BFO heterostructure. Figure S3: Microscale PL modulation of the ML-MoS_2_ driven by the domain-engineered BFO thin film. Figure S4: Field-effect-transistor characteristics of the ML-MoS_2_/PZT heterostructure device. Figure S5: Electric field screening effects of the ML-MoS_2_ on PZT.
