# Supplementary material for: Reconfigurable Local Photoluminescence of Atomically-Thin Semiconductors via Ferroelectric-Assisted Effects
Source: Nanomaterials (Basel). 2019 Nov 15;9(11):1620. doi: 10.3390/nano9111620 (PMC6915559; doi:10.3390/nano9111620)
Supplement: Supplementary file 1 [file nanomaterials-09-01620-s001.pdf]

Supplementary Materials: Reconfigurable Local Photoluminescence of Atomically-Thin Semiconductors via Ferroelectric-Assisted Effects

Changhyun Ko [1,2]

[1] Department of Applied Physics, College of Engineering, Sookmyung Women's University, Seoul 04310, Korea; cko@sookmyung.ac.kr; Tel: +82-2-6325-3184

[2] Institute of Advanced Materials and Systems, Sookmyung Women's University, Seoul 04310, Korea

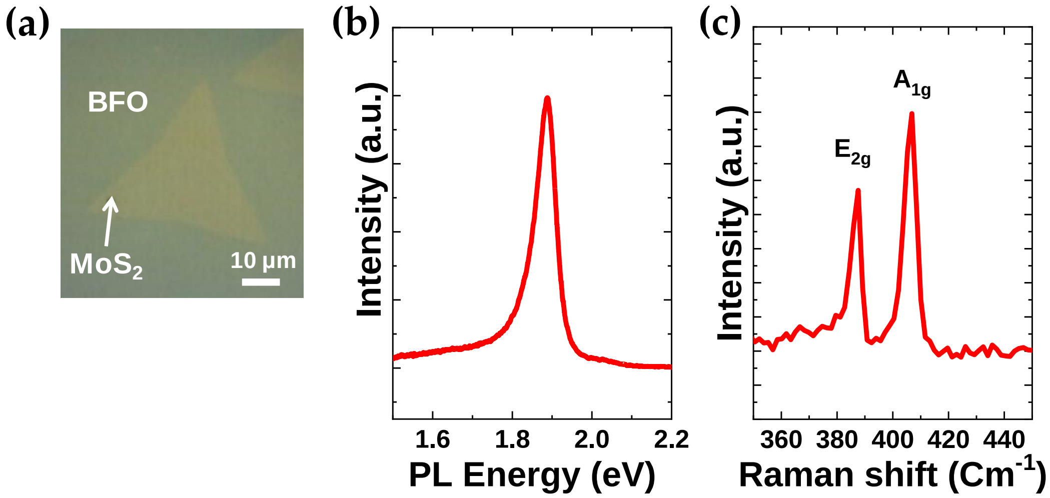

Figure S1. Optical characterization of the ML-MoS_2/BFO heterostructure. (**a**) Optical microscopy image of a representative ML-MoS_2 flake wet-transferred on a BFO thin film surface. (**b**) PL and (**c**) Raman spectra measured from the ML-MoS_2 flake in (**a**).

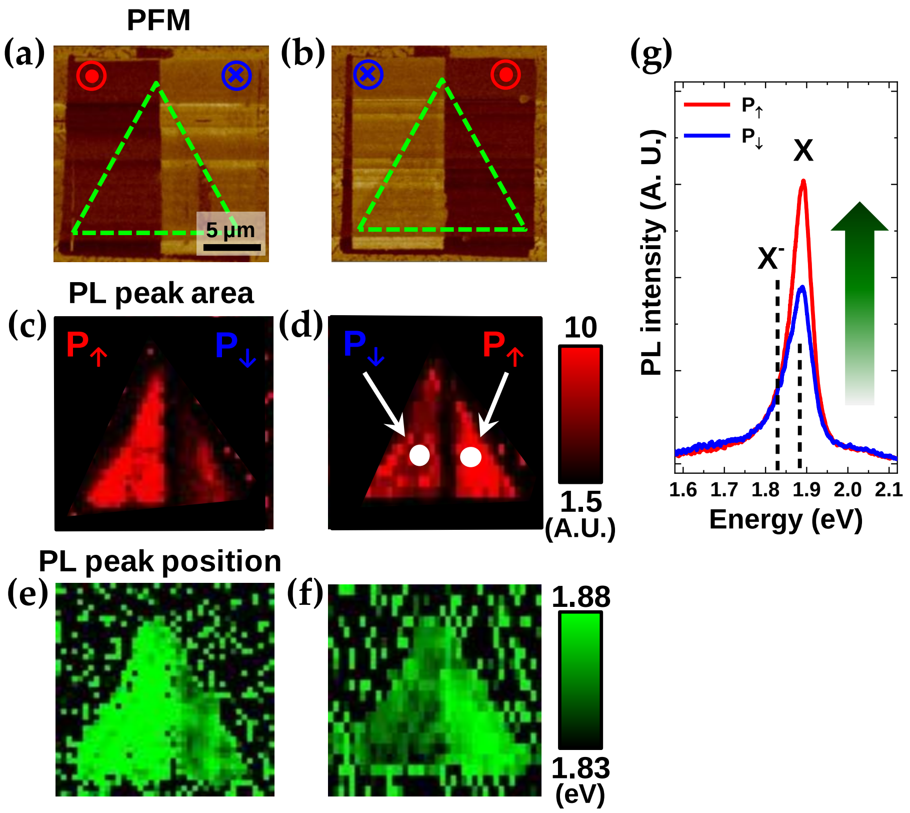

Figure S2. Poling effects on the ML-MoS_2/BFO heterostructure. PFM images of (**a**) the ML-MoS_2 on a BFO thin film whose left and right half areas are polarized in the P↑ and P↓ states by the poling process with V_P of ±12 V, respectively, and (**b**) vice versa. (**c,d**) PL peak area maps of the identical ML-MoS_2 were scanned after poling processes of (a) and (b), respectively. (**e**,**f**) PL peak area maps of the identical ML-MoS_2 were acquired after poling processes of (a) and (b), respectively. (**g**) PL spectra measured from the spots in the P↑ and P↓ regions on the same ML-MoS_2 flake whose positions are marked in (d). X and X^- denote the emissions of neutral and negatively charged excitons, respectively.

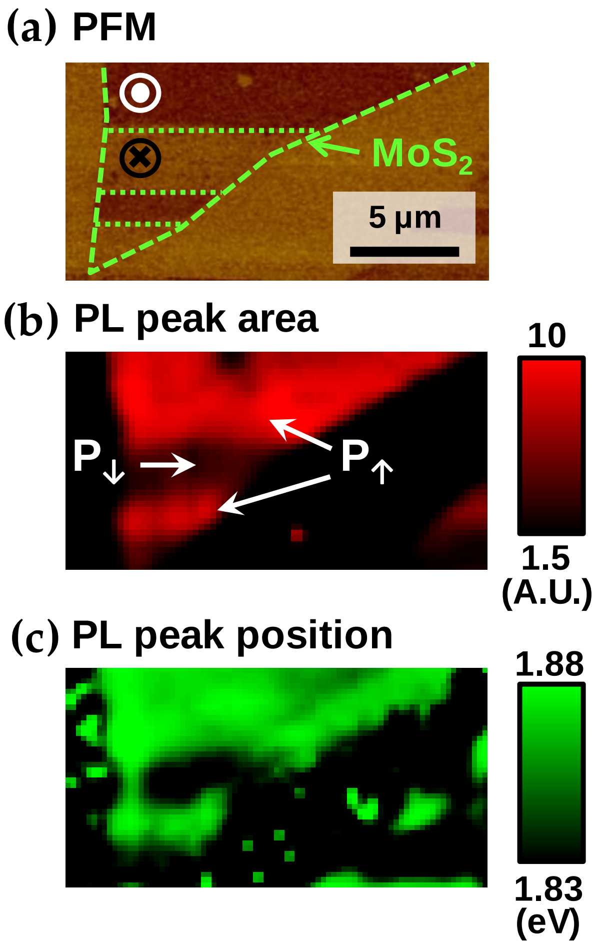

Figure S3. Microscale PL modulation of the ML-MoS_2 driven by the domain-engineered BFO thin film. (**a**) PFM image of the ML-MoS_2 on a BFO thin film poled in the stripe pattern. The bright and dark areas indicate the regions in the P↑ and P↓ states, respectively, and were achieved by applying V_P of ±12 V. Maps of (**b**) PL peak area (**c**) PL peak position of the identical ML-MoS_2 in (a) scanned after poling process.

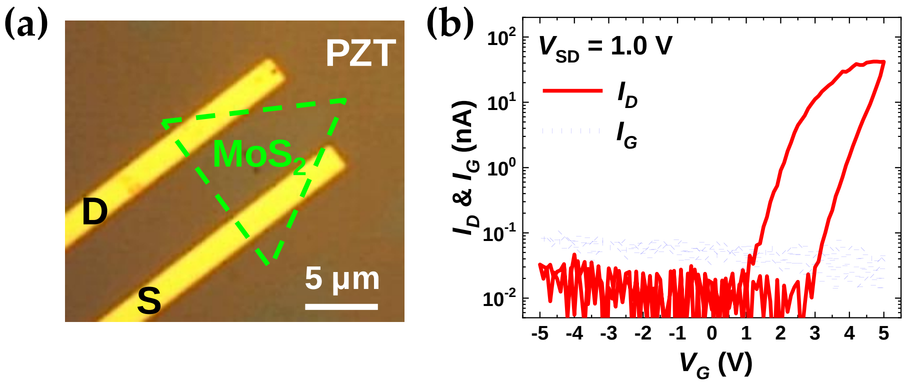

Figure S4. Field-effect-transistor characteristics of the ML-MoS_2/PZT heterostructure device. (**a**) Optical microscopy image of field-effect-transistor (FET) device based on a single ML-MoS_2 flake on a PZT thin film. (**b**) FET characteristic curve of drain current (I_D) vs. gate voltage (V_G) measured at the source-drain voltage (V_{SD}) of 1.0 V. The leakage current (I_G) vs. V_G is also plotted as the dashed line. Note that the absolute values were taken for the negative data values of I_D in the off-state and I_G.

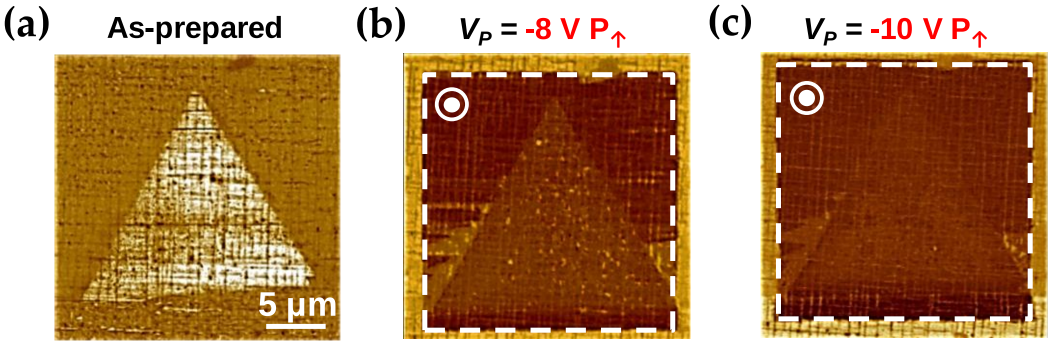

Figure S5. Electric field screening effects of the ML-MoS_2 on PZT. PFM images of a ML-MoS_2 flake on a PZT thin film scanned (**a**) as-prepared before poling and after poling with the V_P of (**b**) -8 V and (**c**) -10 V, respectively. Up to V_P of -8 V, the area of the PZT thin film beneath the ML-MoS_2 flake was not poled fully as can be seen from the many unpoled spots (bright dots) representing that a ML-MoS_2 flake prevents the field penetration into the PZT thin film somehow.
